# Supplementary material for: Automated high-throughput heartbeat quantification in medaka and zebrafish embryos under physiological conditions
Source: Sci Rep. 2020 Feb 6;10:2046. doi: 10.1038/s41598-020-58563-w (PMC7005164; doi:10.1038/s41598-020-58563-w)
Supplement: Supplementary file 1 — Supplementary Information. [file 41598_2020_58563_MOESM1_ESM.pdf]

Supplementary Information

Automated high-throughput heartbeat quantification in medaka and zebrafish embryos under physiological conditions

Jakob Gierten[1,2]*, Christian Pylatiuk[3], Omar Hammouda[2], Christian Schock[3], Johannes Stegmaier[4], Joachim Wittbrodt[2], Jochen Gehrig[5]*, Felix Loosli[6]*

[1]Department of Pediatric Cardiology, University Hospital Heidelberg, Heidelberg, Germany.
[2]Centre for Organismal Studies, Heidelberg University, Heidelberg, Germany.
[3]Institute for Automation and Applied Informatics, Karlsruhe Institute of Technology, Eggenstein-Leopoldshafen, Germany.
[4]Institute of Imaging and Computer Vision, RWTH Aachen University, Aachen, Germany.
[5]ACQUIFER is a division of DITABIS, Digital Biomedical Imaging Systems AG, Pforzheim, Germany.
[6]Institute of Toxicology and Genetics, Karlsruhe Institute of Technology, Eggenstein-Leopoldshafen, Germany.

***Corresponding authors:**
jakob.gierten@med.uni-heidelberg.de, j.gehrig@acquifer.de, felix.loosli@kit.edu

Supplementary Methods

Image pre-processing

To moderately increase the robustness of the automated heart region detection, all acquired images were pseudo-flat-field corrected and intensity normalized by dividing the original unsigned 16-bit TIFF images with a Gauss-filtered (sigma=200) duplicate followed by multiplication with 8000. Corrected images were saved as 16-bit TIFFs and JPEGs. For single mounted embryos, the XY positions of specimen were detected by extracting the center of mass of objects after Gauss filtering (sigma=10) and Otsu thresholding[1]. To reduce image data size, the centre of mass was used to define a bounding box of 600x600 pixels followed by auto-cropping. Parsing of hierarchical data structures was carried out using Perl scripts (HeartBeat_image_preProc; Software) and image processing using ImageJ macros[2,3]. To open videos in the *HeartBeat* software, all frames need to receive (i) time stamps of the format yy.mm.dd_HH.MM.SS,FFF at a given frame rate and (ii) to be listed in subdirectories by well coordinates, for which we used custom MATLAB (Video_to_Image_converter; Software) and Python scripts, respectively. All data and image processing tasks were carried out on an ACQUIFER HIVE system (Ditabis AG, Pforzheim, Germany).

HeartBeat detection and analysis software

The Images are recorded from multiple zebrafish and medaka embryos in a well over time. For further processing, the images are converted to grayscale and saved together with their corresponding time stamps. Detection of the heart(s) and the heartbeat analysis comprises of several sequential steps: segmentation of the regions of interest (ROIs), feature extraction, classification and frequency analysis. The result is presented in a graphical user interface (GUI) and a heatmap. Furthermore, the user can change parameters to optimize the automatic detection procedure. Depending on the changed parameter, the necessary analysis steps are repeated.

Segmentation

The segmentation is performed by accumulating grey value differences between subsequent images. Hereafter, a Gaussian low-pass filter with a user-defined standard deviation of the brightness values within the ROI is applied to smooth the image and to eliminate small segments with high dynamics. Out of different methods for feature

extraction, this method outperformed other methods that we have tested in terms of accuracy and robustness (average values in the image, standard deviation of the brightness values within the ROI, sum of relevant pixel changes in the difference image, number of relevant pixel changes in the difference image, and sum of edges and maximum pixel changes). The standard deviation of the brightness values within the ROI gives a value for how irregular grey values are distributed in the ROI. It does not describe a spatial distribution, but the dispersion of all grey values around the mean value within the ROI. Afterwards, a binary threshold is used to perform the actual segmentation, and the segment with the highest sum of grey values is chosen. Finally, this segment is deleted from the image with accumulated grey values, and this procedure is repeated until a predefined number of segments are identified.

Feature extraction

From every image of a related sequence, the region of the first identified segment is cut, and the standard deviation is calculated with equation (1):

$$\sigma = \sqrt{\frac{1}{|ROI|-1}\sum_{ROI}(x_{u,v} - \bar{x})^2} \quad \text{(eq. 1)}$$

Wherein $|ROI|$ is the pixel number in the heart region, $x_{u,v}$ *the* brightness value at position (u, v) and $\bar{x}$ is the average of all pixel positions within the heart region. Extracting the standard deviation σ of the intensity values for each of the analyzed regions over time allows us to analyze the temporal intensity changes within each potential heart region. These time series of intensity changes exhibit a particular oscillating pattern for regions of the beating heart within a characteristic frequency range and can thus be used to suppress detections in other anatomical regions as detailed in the next section.

Classification

The first step of classification is an analysis of the time series of standard deviations of all identified segments for their frequency components by performing a spectral analysis (Fast Fourier Transformation). This method is used to distinguish whether a ROI is a heart region or not, as movement can also be caused by other moving body parts such as fins or blood movement in larger blood vessels. By defining upper and

lower limits for the expected heart rates the frequency band of the spectral analysis can be limited to this range. The highest peak of a frequency in the resulting spectrum of a ROI is most likely the heart region. Subsequently, a ranking for the other segmented ROIs is performed according to the frequency peaks and a predefined number of segments are classified as a heart region. Blood flow in the tail region typically show lower amplitude peaks than the heart region ROI and fin movements typically show amplitude peaks at lower frequencies than the typical frequency range of a fish heart in the spectral analysis.

Heart frequency analysis

In order to determine the beat-to-beat intervals of a time series, the repetitive maximum values of the feature "standard deviation of segment brightness" are used. Peak values that do not exceed a predefined threshold are removed and the time stamps of the corresponding images are saved to a file. The average heart frequency f_{heart} can be calculated from the reciprocal value of the mean of all beat-to-beat intervals as given in equation (2):

$$f_{heart} = \frac{1}{(\frac{1}{n}\sum_{i=1}^{n} RR_i)} \quad (2)$$

Where n is the number of calculated beat-to-beat intervals and RR the length of a beat-to-beat interval. Additionally, arrythmicity (heart rate variability) is evaluated by calculating the square root of the mean of the sum of the squares of the successive differences between consecutive beat-to-beat intervals (RMSSD). The corresponding equation is given in equation (3) in accordance with the recommendations of the Task Force of The European Society of Cardiology and The North American Society of Pacing and Electrophysiology[4].

$$RMSSD = \sqrt{\frac{\sum_{i=1}^{n-1}(RR_i - RR_{i+1})^2}{n-1}} \quad (3)$$

Graphical User Interface (GUI)

After starting the software, the parent folder containing the subfolders with the image sequences has to be chosen and the number of fish to be analysed has to be specified.

After some seconds of loading and processing the images an interactive window pops up (Fig. 2).

Supplementary Figures

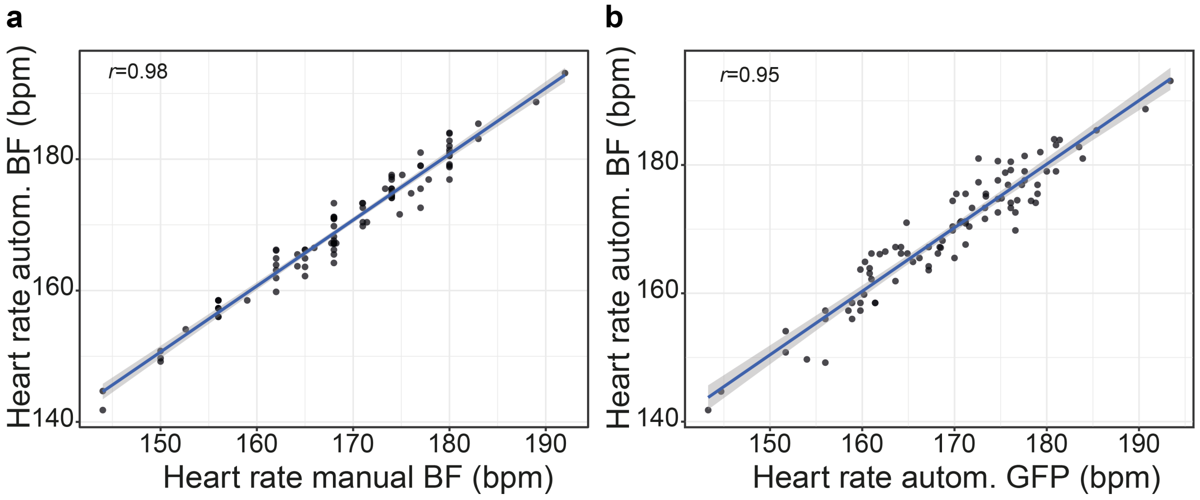

Supplementary Figure S1. Comparisons of manually and automatically quantified heart rates using medaka embryos. (**a,b**) To assess reproducibility of heart rate scores performing manually counting or software-assisted readout of heart rates in bright-field (BF) and eGFP channels, medaka *myl7::eGFP* embryos were imaged for 10 s with 13 fps. (**a**) Using bright-field image sequences, correlation analysis between manually counted heart rates and heart rates scored with the *HeartBeat* software yielded a Pearson correlation coefficient *r*=0.98 (n=91). (**b**) Comparing heart rates derived from eGFP and BF channel image sequences quantified with the *HeartBeat* software, showed a correlation of *r*=0.95 (n=91).

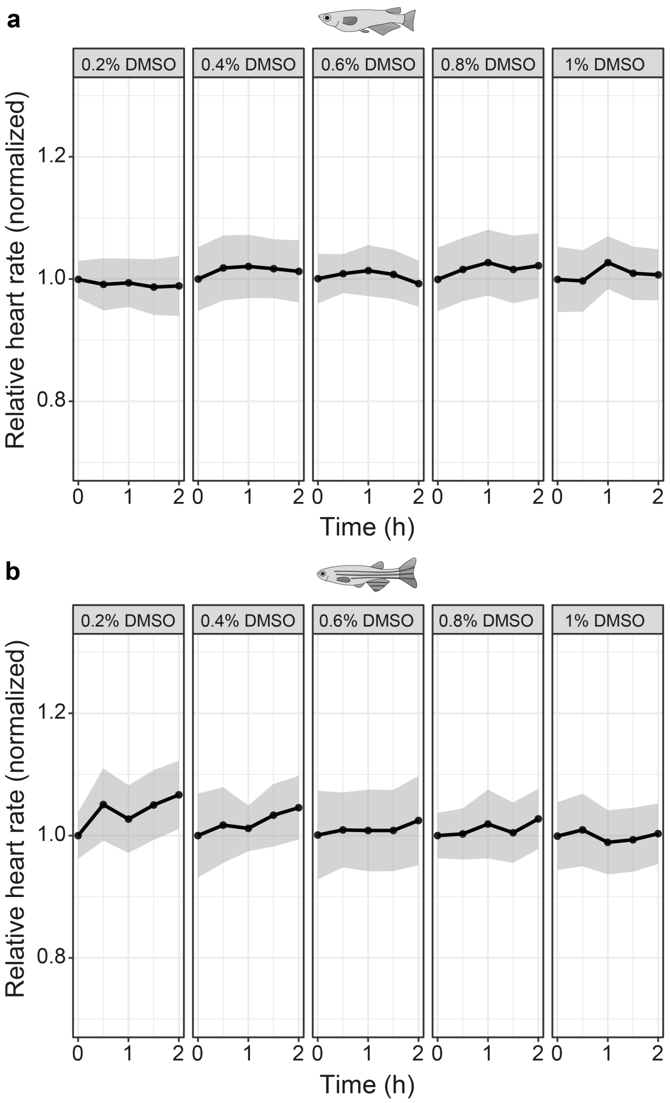

Supplementary Figure S2. DMSO titration on medaka and zebrafish embryos. (**a,b**) Differences between all DMSO concentrations over time and between DMSO concentrations at corresponding time points were tested with one-way ANOVA and pairwise comparisons using Student's *t*-test. DMSO titration (0.2-1%) on medaka embryos (102-104 hpf) revealed no significant changes of heart rate within 2 h (**a**, *n*=16 for each DMSO concentration and ERM). In zebrafish (32-34 hpf), heart rates exhibited moderate increase at lower DMSO concentrations and reached formal significant differences between 0.2% DMSO baseline vs. 2 h and between 0.2% DMSO and 1% DMSO after 2 h incubation ($P<0.05$, respectively; **b**, *n*=8-16 for each DMSO concentration and E3); data is shown as mean±s.d. At all points in time, each heart rate measurement is normalized to the corresponding ERM/E3 mean to account for stage-dependent changes and is expressed relative to the corresponding baseline group mean (time point 0).

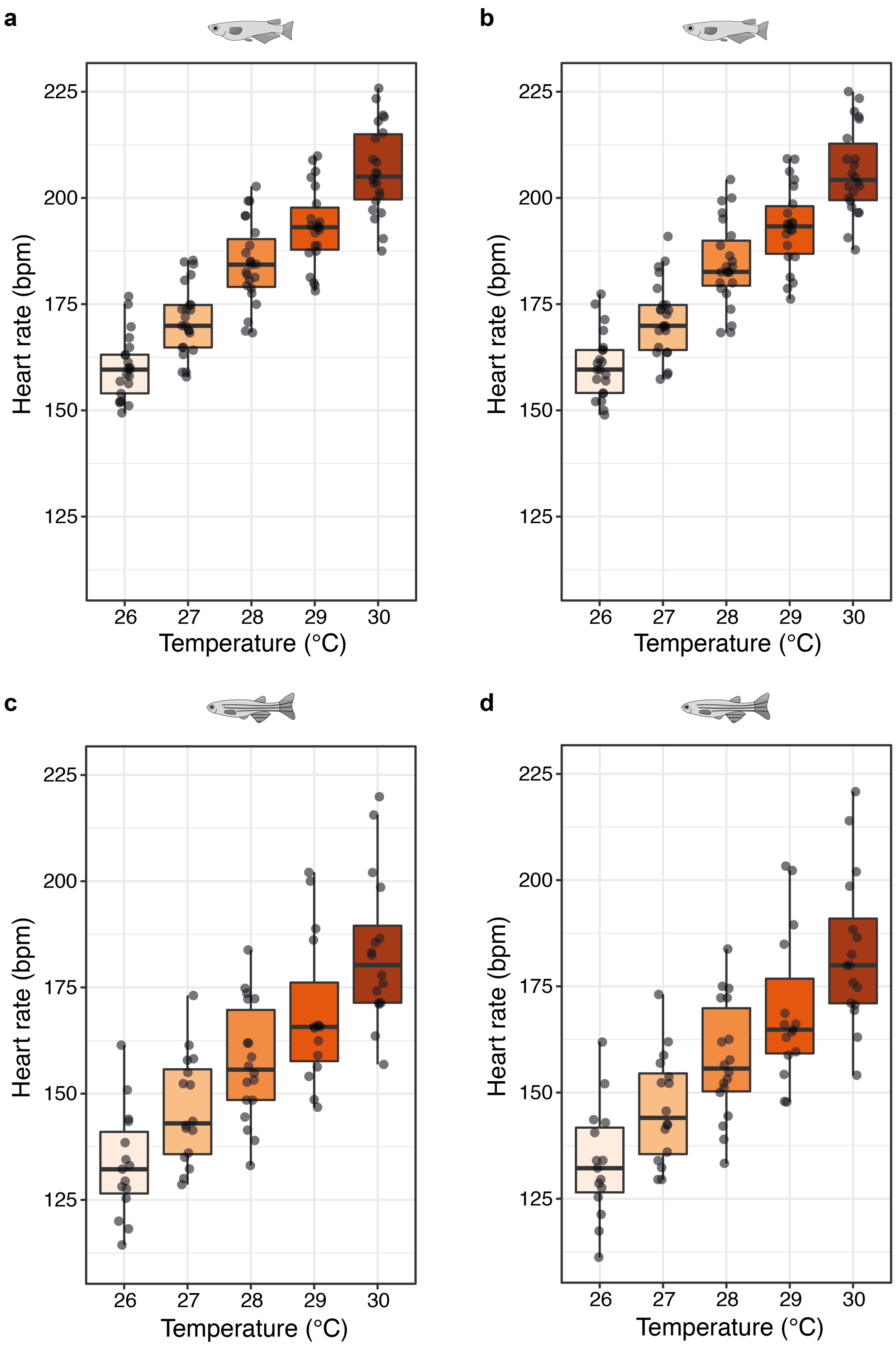

Supplementary Figure S3. Comparable sensitivity of the heartbeat assay at 30 fps and synthetic 15 fps sampling rates. Heart rate response to temperature gradient (26-30°C) in medaka embryos (103-105 hpf) recorded at 30 fps (a) and derived synthetic 15 fps data (b); *n*=21-23 at each temperature. Equivalent experiment for zebrafish embryos (31-33 hpf) comparing 30 fps (c) to synthetic 15 fps (d) data; *n*=15-18 at each temperature. Heart rate quantification yielded similar results for both real 30 fps and

synthetic 15 fps data. This finding was validated in a biological replicate (data not shown). Significant differences between heart rates at each temperature were tested with one-way ANOVA and pairwise comparisons using Student's t-test: *P*-values for Supplementary Figure S3 are listed in Supplementary Tables S3-6.

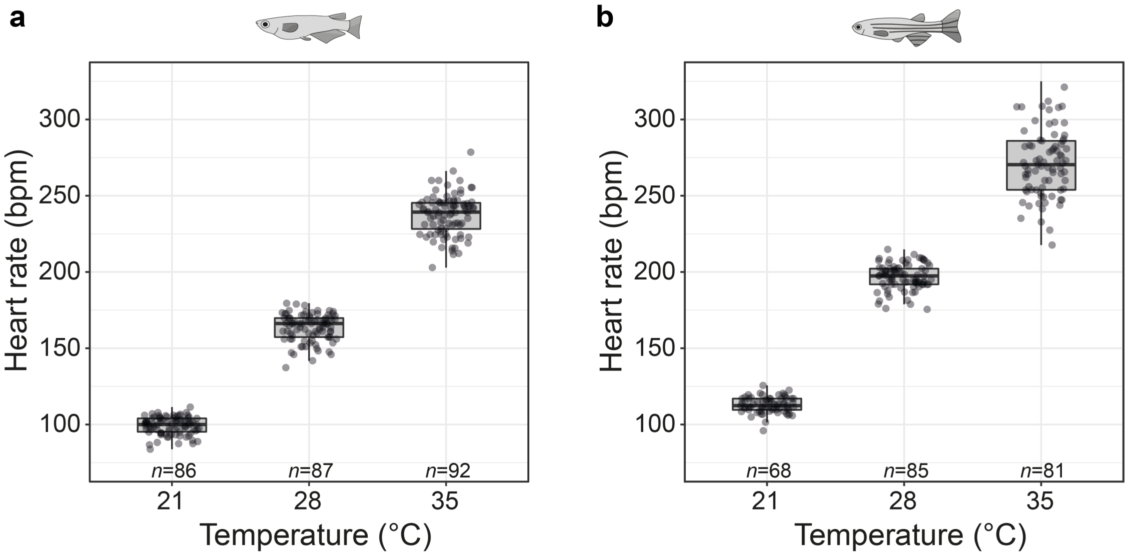

Supplementary Figure S4. Embryonic heart rates at 21°C, 28°C, and 35°C. (**a,b**) Heart rate response to temperature ramps in medaka (100-102 hpf, n=86-92 at each temperature in **a**) and zebrafish (36-38 hpf, n=68-85 at each temperature in **b**). Data is given as box plots (median±interquartile range) and overlaid scatter plots of original heart rate values.

Supplementary Tables

Supplementary Table S1: *P*-values to Figure 5a, temperature gradient for medaka embryos recorded at 13 fps.

	26°C	27°C	28°C	29°C
27°C	0.01583	-	-	-
28°C	< 2e-16	1.2e-15	-	-
29°C	< 2e-16	< 2e-16	0.00052	-
30°C	< 2e-16	< 2e-16	< 2e-16	5.7e-09

Supplementary Table S2: *P*-values to Figure 5b, temperature gradient for zebrafish embryos recorded at 13 fps.

	26°C	27°C	28°C	29°C
27°C	9.0e-08	-	-	-
28°C	< 2e-16	3.4e-13	-	-
29°C	< 2e-16	< 2e-16	0.01	-
30°C	< 2e-16	< 2e-16	1.1e-14	1.6e-06

Supplementary Table S3: *P*-values to Supplementary Figure S3a, temperature gradient for medaka embryos recorded at 30 fps.

	26°C	27°C	28°C	29°C
27°C	0.00091	-	-	-
28°C	1.7e-13	2.9e-05	-	-
29°C	< 2e-16	1.3e-11	0.01996	-
30°C	< 2e-16	< 2e-16	1.1e-11	4.0e-05

Supplementary Table S4: *P*-values to Supplementary Figure S3b, temperature gradient for medaka embryos at synthetic 15 fps.

	26°C	27°C	28°C	29°C
27°C	0.0023	-	-	-
28°C	1.7e-12	6.7e-05	-	-
29°C	< 2e-16	3.8e-11	0.0212	-
30°C	< 2e-16	< 2e-16	3.7e-11	9.9e-05

Supplementary Table S5: *P*-values to Supplementary Figure S3c, temperature gradient for zebrafish embryos recorded at 30 fps.

	26°C	27°C	28°C	29°C
27°C	0.19121	-	-	-
28°C	0.00020	0.36699	-	-
29°C	8.9e-08	0.00078	0.30371	-
30°C	4.2e-13	8.9e-09	2.6e-05	0.08218

Supplementary Table S6: *P*-values to Supplementary Figure S3d, temperature gradient for zebrafish embryos at synthetic 15 fps.

	26°C	27°C	28°C	29°C
27°C	0.20986	-	-	-
28°C	0.00022	0.35209	-	-
29°C	9.1e-08	0.00070	0.29315	-
30°C	9.8e-13	1.8e-08	5.3e-05	0.13660

Legends for Videos files

Video S1 and S2. 2:1 AV block in medaka and zebrafish after terfenadine treatment. Video 1 (medaka) and video 2 (zebrafish) show representative examples of embryos treated with 100 µM terfenadine inducing 2:1 AV block (recorded with 4x objective).

Software

Scripts and *HeartBeat* software are available through https://osf.io/kygd4/

(1) Perl script (HeartBeat_image_preProc) executable for Windows to generate an ImageJ macro for image pre-processing applicable to image sequences with single embryo per well (Image pre-processing; Detailed Methods).
(2) MATLAB script (Video_to_Image_converter) to add time stamps of the format yy.mm.dd_HH.MM.SS,FFF to single frames.
(3) *HeartBeat* software.
(4) User manual for *HeartBeat* software.

Supplementary References

1. Otsu, N. A Threshold Selection Method from Gray-Level Histograms. *IEEE Transactions on Systems, Man, and Cybernetics* **9,** 62–66 (1979).
2. Schneider, C. A., Rasband, W. S. & Eliceiri, K. W. NIH Image to ImageJ: 25 years of image analysis. *Nat. Methods* **9,** 671–675 (2012).
3. Schindelin, J. *et al.* Fiji: an open-source platform for biological-image analysis. *Nat. Methods* **9,** 676–682 (2012).
4. Task Force of the European Society of Cardiology and the North American Society of Pacing and Electrophysiology. Heart rate variability: standards of measurement, physiological interpretation and clinical use. *Circulation* **93,** 1043–1065 (1996).
